# Effect of Side Chain Substituent Volume on Thermoelectric Properties of IDT-Based Conjugated Polymers

**DOI:** 10.3390/molecules26040963

**Published:** 2021-02-11

**Authors:** De-Xun Xie, Tong-Chao Liu, Jing Xiao, Jing-Kun Fang, Cheng-Jun Pan, Guang Shao

**Affiliations:** 1 School of Chemistry, Sun Yat-sen University, Guangzhou 510275, China; xiedx9@mail.sysu.edu.cn (D.-X.X.); xiaoj69@mail.sysu.edu.cn (J.X.); 2 Shenzhen Research Institute, Sun Yat-sen University, Shenzhen 518057, China; 3 Shenzhen Key Laboratory of Polymer Science and Technology, College of Materials Science and Engineering, Shenzhen University, Shenzhen 518060, China; liu21645@163.com (T.-C.L.); pancj@szu.edu.cn (C.-J.P.); 4 School of Chemical Engineering, Nanjing University of Science and Technology, Nanjing 210094, China

**Keywords:** organic thermoelectric materials, conjugated polymers, power factor

## Abstract

A p-type thermoelectric conjugated polymer based on indacenodithiophene and benzothiadiazole is designed and synthesized by replacing normal aliphatic side chains (**P1**) with conjugated aromatic benzene substituents (**P2**). The introduced bulky substituent on **P2** is detrimental to form the intensified packing of polymers, therefore, it hinders the efficient transporting of the charge carriers, eventually resulting in a lower conductivity compared to that of the polymers bearing aliphatic side chains (**P1**). These results reveal that the modification of side chains on conjugated polymers is crucial to rationally designed thermoelectric polymers with high performance.

## 1. Introduction

Organic thermoelectric materials (OTEs) (including semiconducting molecules and polymers) are becoming active materials because of the prospect of realizing lightweight, low cost, and wearable thermoelectric generators [1,2,3,4,5]. π-conjugated polymers have been widely studied as active layers in organic electronics (e.g., polymer solar cells [6], organic field-effect transistors [7], sensors [8]). The study of π-conjugated polymers as thermoelectric materials has also captured the attention of researchers owing to their unique advantages compared to inorganic materials and organic small molecules [9]. Since the astonishing thermoelectric performance of PEDOT has been reported [10,11], a variety of semiconducting polymers have been selected as the candidates for thermoelectric properties investigations [12,13,14,15].

It has been revealed that the chemical structures of the polymers and appropriate doping are the key factors to determine their thermoelectric performances, through optimizing the dimensionless figure of merit value *ZT* = S^2^σT/κ, where σ, S, κ, and T are the electrical conductibility, the Seebeck coefficient, the thermal conductivity, and absolute temperature [16,17]. Up to now, there is still no clear guidance to design semiconducting polymers with high thermoelectric performance, although a number of organic thermoelectric materials have been developed and some structure–properties relationships could be obtained from the reported results. 

The incorporation of side chains to the conjugated backbones of conjugated polymers (CPs) is a popular strategy to tune the solubility and interchain packing of CPs in the field of organic electronics [18,19]. CPs with donor (D)–acceptor (A) backbone usually have narrow bandgaps and show high charge carrier mobility, which is beneficial to large Seebeck coefficients and electrical conductivities [20,21,22]. Wang et al. developed a series of thermoelectric-conjugated polymers with donor–acceptor structures and found that the polymer backbone structure greatly influences their thermoelectric properties [23,24,25,26]. Pei et al. recently found that the electron-withdrawing modification of the donor units in the D-A conjugated polymer can enhance the electron affinity of the polymer and change the polymer packing orientation, eventually leading to the substantially improved miscibility and doping efficiency [27]. Zhu et al. noticed that a selenium-substituted DPP polymer exhibited a significant high figure of merit (*ZT*) value of 0.25 representing the highest value for p-type high-mobility conjugated polymers [28]. Lei et al. developed a pyrazine-flanked diketopyrrolopyrrole (DPP), which showed the highest power factor among all the reported solution-processable n-type conjugated polymers [29]. Not only the backbone modification but also the alternation of side chains can greatly influence the thermoelectric performance of conjugated polymers. Woo et al. replaced the normal alkyl side chains with bis(alkylsulfanyl)methylene side chains on the cyclopentadithiophene (CPDT)-based donor–acceptor semiconducting polymers and found that the sp^2^-hybridized olefinic bis(alkylsulfanyl)methylene side chains and the sulfur–sulfur (S-S) chalcogen interactions can extend a chain planarity with strong interchain packing, which provided an enhanced thermoelectric performance [30]. 

We systematically investigated the influence of two-dimensional conjugated side chains on the thermoelectric performance of benzodithiophene (BDT)-based conjugated polymers and found that the polymers with conjugated side chains exhibited much larger power factors (101.3 μW m^−1^K^−2^, 350 K) compared to that of the polymers with aliphatic side chains (0.9 μW m^−1^K^−2^, 350 K) under the same doping condition [31]. In order to expand our design strategy to other conjugated polymers, herein, we prepared two indacenodithiophene (IDT) and benzothiadiazole (BT)-based semiconducting polymers bearing normal alkyl side chains (**P1**) and side chains with conjugated aromatic benzene ring (**P2**), and we found that the larger size aromatic side chains on **P2** reduced the interchain interactions, thus resulting relatively lower electrical conductivities as well as lower thermoelectric performances. These results indicate that the introduction of aromatic conjugated side chain does not always result in higher thermoelectric performance, and many other factors also need to be taken into considerations.

## 2. Experimental Section

### 2.1. General

^1^H-NMR spectra of the polymers were recorded on a Bruker AVANCE III operating at 600 MHz at room temperature in CDCl_3_. Thermal gravimetric analysis (TGA) was performed on a TGA-55 instrument (TA Instruments, New Castle, DE, USA) from room temperature to 700 °C under a nitrogen flow of 20 mL min^−1^ with a heating rate of 10 °C min^−1^. Gel permeation chromatography (GPC) (Waters, Milford, MA, USA) was used to determine the molecular weights and polydispersity index (PDI) of the polymers using THF as the eluent and polystyrene as a standard. Differential scanning calorimetry (DSC) was carried out on a DSC7020 instrument (Hitachi, Tokyo, Japan) in a temperature range from 0 to 300 °C at a heating rate of 10 °C min^−1^. Ultraviolet–visible–near-infrared (UV–VIS-NIR) absorption spectra were performed on a Lambda 950 spectrophotometer (PerkinElmer, Hopkinton, MA, USA). A CHI 660E electrochemical workstation (CH Instruments, Austin, TX, USA) was used to do cyclic voltammetry (CV) characterizations. Tapping-mode atomic force microscopy (AFM) images of polymer films were obtained using AFM (Bruker Dimension ICON, Billerica, MA, USA) to observe the roughness of the films surface. Grazing incidence X-ray diffraction (GI-XRD) was determined using a SmartLab X-ray diffractometer (Rigaku, Tokyo, Japan) with a copper target (*λ* = 1.54 Å), and the incident range was 2–40°. The electrical conductivity (σ) and Seebeck coefficient (S) of the polymer films were recorded using the commercialized thermoelectric test system (MRS-3) (Wuhan Joule Yacht Science & Technology, China).

### 2.2. Synthesis of the Copolymers

General procedure for the synthesis of **P1**: A mixture of 2,7-dibromo-4,4,9,9-tetraoctyl-4,9-dihydro-*s*-indaceno[1,2-*b*:5,6-*b*′]dithiophene (0.200 g, 0.229 mmol), bis(trimethylstannyl) compound (0.143 g, 0.229 mmol), Pd_2_(dba)_3_ (0.010 g, 0.011 mmol), and P(*o*-tol)_3_ (0.017 g, 0.057 mmol) in anhydrous chlorobenzene (5 mL) with a nitrogen flow was sealed and stirred for 72 h at 110 °C. After the mixture was cooled to room temperature, the polymer was precipitated by the addition of excess methanol. The precipitate was sequentially washed with methanol, acetone, and deionized water. After drying under vacuum, the polymer was obtained as black powder.

**P1**: black powder, 84.3 %, ^1^H-NMR (600 MHz, TMS), δ/ppm 0.80–0.87 (t, 12H, CH_2_-CH_3_), 0.88–1.51 (m, 48H, CH_2_), 1.94 (d, 8H, CH_2_), 7.22 (s, 2H, aromatic), 7.27–7.47 (m, 4H, thiophene), 7.92 (s, 2H, thiophene), 8.08 (s, 2H, aromatic) (Figure S1).

**P2**: black powder, 94.7 %, ^1^H-NMR (600 MHz, TMS), δ/ppm 0.74–0.99 (t, 12H, CH_2_-CH_3_), 1.05–1.50 (m, 48H, CH_2_), 2.53 (d, 8H, CH_2_), 6.97–7.55 (m, 22H, aromatic), 7.82 (s, 2H, aromatic), 8.00 (s, 2H, aromatic) (Figure S1).

### 2.3. Thin Films Preparation

The stock solutions of **P1** (5 mg mL^−1^) and **P2** (5 mg mL^−1^) in chlorobenzene have been prepared via ultrasonication to form homogeneous solutions, then the stock solution was drop-casted onto the precleaned glass slides to produce polymer thin films. In the different vials, FeCl_3_ was dispersed in acetonitrile with vigorous stirring to form 0.1 M homogeneous solutions. The as-prepared thin films were then immersed into the FeCl_3_ solutions with different doping times, and then, the doped thin films are used for testing.

## 3. Results and Discussions

### 3.1. Polymer Design, Synthesis, and Characterizations

**Scheme 1:**
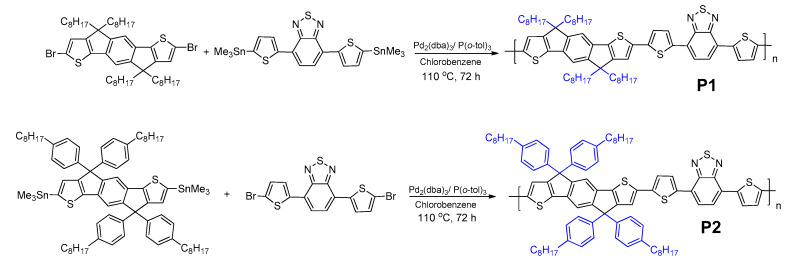
Synthetic routes of **P1** and **P2**.

Scheme 1 illustrates the chemical structures of the IDT- and BT-based copolymers with octyl side chain (**P1**) and conjugated side chain by inserting the aromatic benzene ring between the IDT backbone and octyl (**P2**). Both of them have been prepared via Stille coupling reaction using Pd_2_(dba)_3_ as a catalyst in chlorobenzene. The detailed synthetic procedures have been described in the experimental section. By size exclusion chromatography (SEC) with tetrahydrofuran (THF) eluent at 25 °C, the number-average molecular weight (*M*_n_) was measured to be 58.5 kDa (polydispersity index (PDI) = 2.3) and 87.1 kDa (PDI = 1.9) for **P1** and **P2**, respectively (Table 1 and Figures S2 and S3). The copolymers show good solubility in common organic solvents (chlorobenzene, o-dichlorobenzene, chloroform, and toluene). The thermal properties of **P1** and **P2** are investigated by thermogravimetric analysis (TGA) and differential scanning calorimetry (DSC) measurements. We noticed that **P1** and **P2** show high decomposition temperatures (*T*_d_) with 5% weight loss at 401 °C and 392 °C, respectively, indicating that the two polymers have high thermal stability (Table 1 and Figure S4). The glass transition temperatures for **P1** and **P2** are 133 and 203 °C, respectively, indicating that the introduction of aromatic benzene ring to the side chain rigidified the polymer backbone of the IDT-based copolymers. There are no obvious thermal transitions for both polymers in the DSC measurements in the range of 0–300 °C (Figure S5), indicating that both of them are amorphous polymers.

Density functional theory (DFT) (Spartan 2019, B3LYP/6-31G** level) calculations were performed on the dimer of the polymers to understand the geometric and electronic structures of **P1** and **P2**. All the alkyl side chains have been replaced with methyl groups for clarity. The top and side views of the computation models with the minimal energy level are shown in Figure S6. The two polymers exhibited relative planar backbones, which should be beneficial for the transporting of charger carriers along the polymer chains. **P1** and **P2** exhibited similar molecular orbital distributions, and the highest occupied molecular orbitals (HOMO) are delocalized over all the aromatic units (IDT and BT units), whereas the lowest unoccupied orbitals (LUMO) are mainly delocalized at the BT moiety. The values of HOMO and LUMO are calculated to be −4.6 and −2.7 eV, respectively, for both **P1** and **P2** (Figure S6). Although the molecular orbitals and energy levels of the dimers of **P1** and **P2** are identical, the geometries at the substitution point for **P1** and **P2** are different, the two aromatic substituted benzene rings on **P2** are perpendicular to the IDT backbone to form a shish-kebab structures, which might hinder the intense π–π* stacking of the polymer backbones. The electrical conductivities of **P2** are expected to be lower than those of **P1** under the same doping conditions.

**Figure 1:**
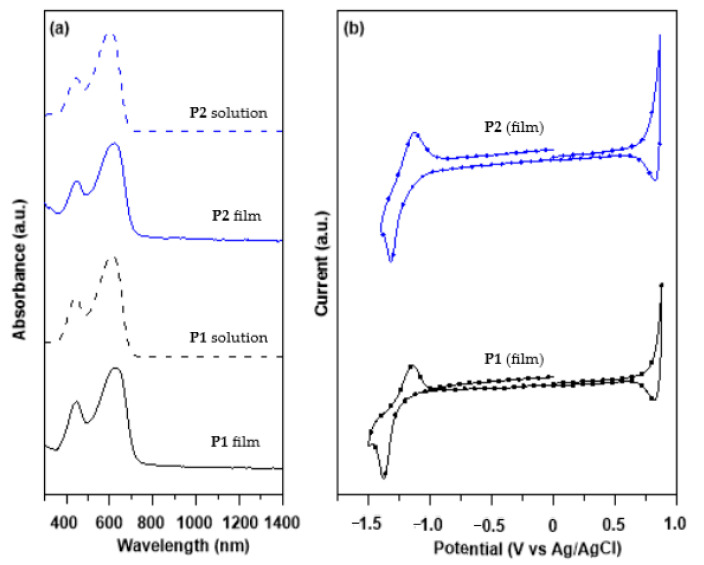
(**a**) Ultraviolet–visible–near-infrared (UV–VIS–NIR) absorption spectra of nondoped **P1** and **P2** in film states (solid line) and solutions (dotted line). (**b**) Cyclic voltammograms of nondoped **P1** and **P2** in drop-casted films.

Figure 1a shows the normalized UV–VIS–NIR absorption spectra of **P1** and **P2** in film states and chloroform. In the solutions, **P1** and **P2** showed broad absorption from 400 to 800 nm, and the absorption peak at high energy level should be attributed to the π–π* and the low energy peak could be originated from the intramolecular charge transfer (ICT) transitions [31]. The absorption maxima (*λ_abs_*) in chloroform were observed to be 604 and 614 nm for **P1** and **P2**, respectively. The absorption maxima (*λ_abs_*) in film states for **P1** and **P2** are determined to be 620 and 630 nm, respectively. From the solutions to film states, the absorption maxima both shifted around 16 nm for the two polymers, which should be generated from the conformation change of backbones upon film formation. Based on the onset of the absorption spectra in the film states, the optical band gaps for **P1** and **P2** are determined to be 1.70 and 1.68 eV, respectively.

Cyclic voltammetry (CV) measurements were performed to determine the HOMO/LUMO frontier orbital energy levels, as shown in Figure 1b, the HOMO/LUMO values were −5.70/−3.80 eV for **P1** and −5.73/−3.91 eV for **P2**, respectively. Both of them showed low HOMO levels, which can improve the p-type doping (electrons transfer from the polymer backbones to the dopant) of the polymer backbones.

### 3.2. Morphology Study

**Figure 2:**
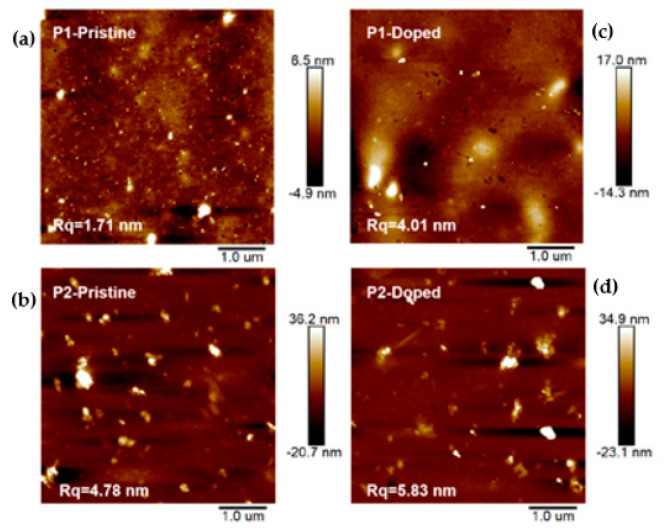
AFM images of **P1** (**a**) and **P2** (**b**) pristine films and films doped with FeCl_3_ (0.1 M) for 15 min, **P1** (**c**) and **P2** (**d**).

The microstructures of **P1** and **P2** thin films were investigated by atomic force microscopy (AFM) as shown in Figure 2, the AFM topography images of undoped **P1** and **P2** films exhibited relatively smooth surfaces. A few particles in the images might be due to the palladium residues introduced from the synthesis, which is extremely difficult to be completely removed though we did thorough purification. Upon doping the polymer films with FeCl_3_ (0.1 M) for 15 min, the roughness of the two polymer films was slightly increased with the root-mean-square (RMS) enhancing from 1.71 to 4.01 nm for **P1** and RMS enhancing from 4.78 to 5.83 nm for **P2**. The morphology of the polymer film after doping is still smooth and flat, and there is no phenomenon of dopant aggregation, which can also be proved from SEM and EDS tests (Figure S7). The AFM phase images shown in Figure S8 also confirmed the smooth surfaces of the polymer films before and after doping with FeCl_3_.

**Figure 3:**
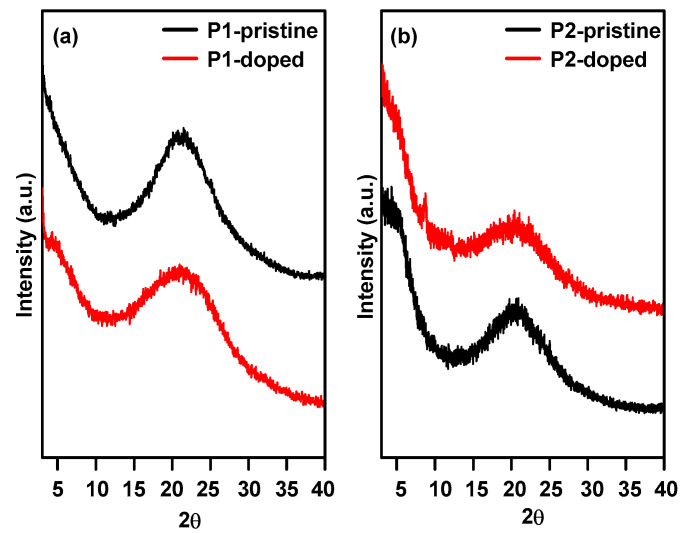
Grazing incidence X-ray diffraction (GI-XRD) curves of polymer films **P1** (**a**) and **P2** (**b**) with pristine condition and doped with FeCl_3_ (0.1 M) for 15 min.

In order to deepen our understanding of the nanostructures of neat and doped films (**P1** and **P2**), we conducted grazing incidence wide-angle X-ray scattering (GI-XRD) experiments as shown in Figure 3. As concluded from the DSC results, GI-XRD diffractograms also confirmed that both **P1** and **P2** showed amorphous features in the thin-film states with a halo diffraction between 15° and 30°, and the widths of diffraction peaks for pristine and doped films of **P1** are narrower than those of diffraction peaks for **P2**, suggesting that interchain packing of **P1** is much stronger that of **P2**. After doping with FeCl_3_, the widths of the diffraction peaks are much larger than those of peaks for pristine films, indicating that the addition of dopant disturbed the interchain packing of conjugated polymer backbones.

### 3.3. Characterizations of the Doped Conjugated Polymers

**Figure 4:**
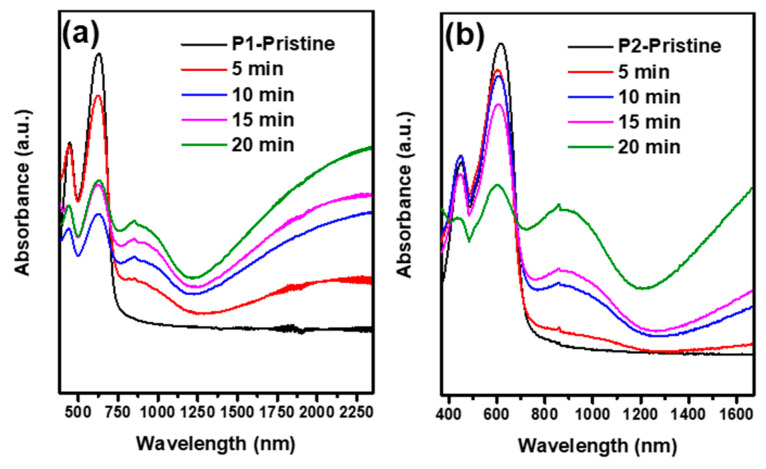
The time dependence of normalized UV–VIS–NIR absorption spectra of polymer films **P1** (**a**) and **P2** (**b**) doped with FeCl_3_.

In order to improve the electrical conductivity, p-doping was performed with a chemical dopant (FeCl_3_). The polymer thin films were immersed into the FeCl_3_ solutions in acetonitrile for different times, and the UV–VIS–NIR spectra of doped **P1** and **P2** were recorded with increasing the doping time as shown in Figure 4. The newly appeared peaks at around 854 nm for **P1** and 856 nm for **P2** could be assigned to the formation of polaron states, and the intensities are increased in compliance with increasing the doping time. The calculated intensities of *λ*_854_/*λ*_620_ for **P1** and *λ*_856_/*λ*_630_ for **P2** are also gradually increased as increasing the doping time (Figure S9). The intensities of the new peaks over 1200 nm for the doped films of **P1** and **P2** exhibited the similar trend as before, which could be attributed to the formation of bipolarons. These results indicated that FeCl_3_ is an efficient dopant for **P1** and **P2**, and the difference of side chains has no effect on the doping behavior of the semiconducting polymers.

**Figure 5:**
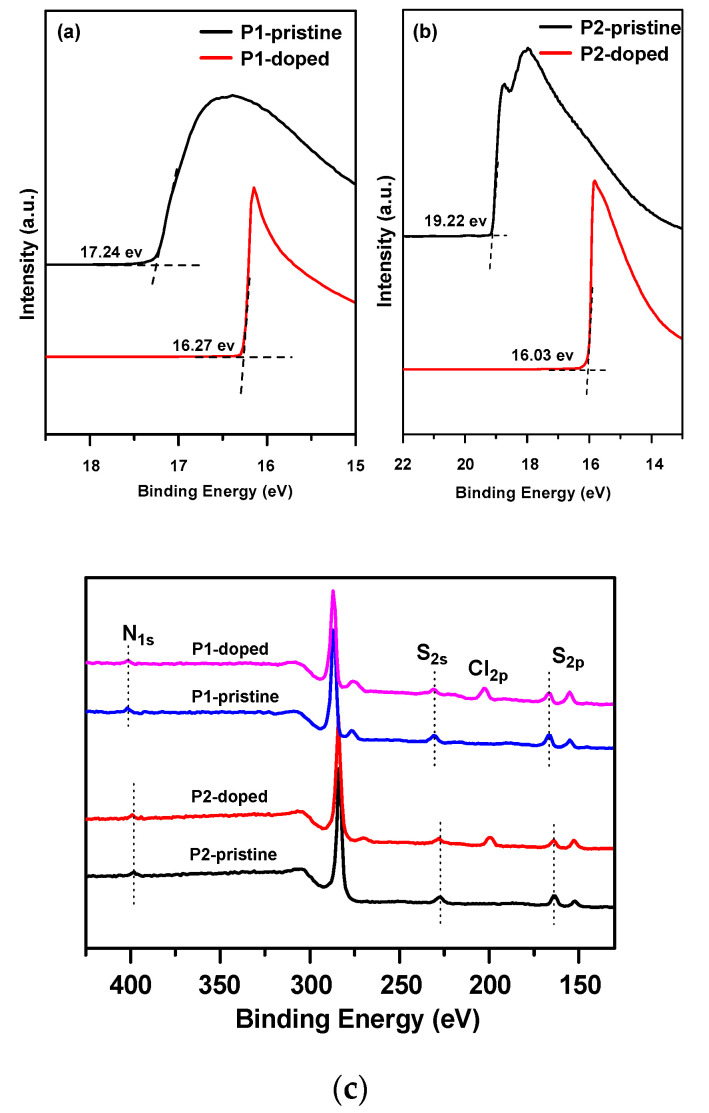
Ultraviolet photoelectron spectroscopy (UPS) spectra (He I α radiation, photon energy = 21.22 eV) showing the secondary electron cut-off region for **P1** (**a**) and **P2** (**b**) in pristine and films doped with FeCl_3_ (0.1 M) for 15 min. XPS spectra (**c**) S 2_S_ and S 2_P_ curves of pristine polymer films and films doped with FeCl_3_ (0.1 M) for 15 min.

The ultraviolet photoelectron spectroscopy (UPS) measurements (Figure 5) were performed to support the UV–VIS–NIR spectra results. The second electron cutoff of **P2** shifted by 3.19 eV from the pristine state (16.03 eV) to doped state (19.22 eV), which is much larger than the shift of **P1** (0.97 eV); the upward shift of the spectra indicated the generation of hole carriers and the effective P-type doping of both **P1** and **P2**, and **P2** is more readily p-doped with FeCl_3_ than that of **P1**. The X-ray photoelectron spectroscopy (XPS) spectra of the pristine and doped films are shown in Figure 5c. The XPS N(1s) spectra (around 400 eV), S(2s) spectra (around 225 eV), and S(2p) (around 170 eV) are identical for both **P1** and **P2**. The newly appeared peak at around 202 eV should be attributed to the Cl_2p_ spectrum of the dopant (FeCl_3_), indicating that the FeCl_3_ can successfully dope the semiconducting polymer backbones.

**Figure 6:**
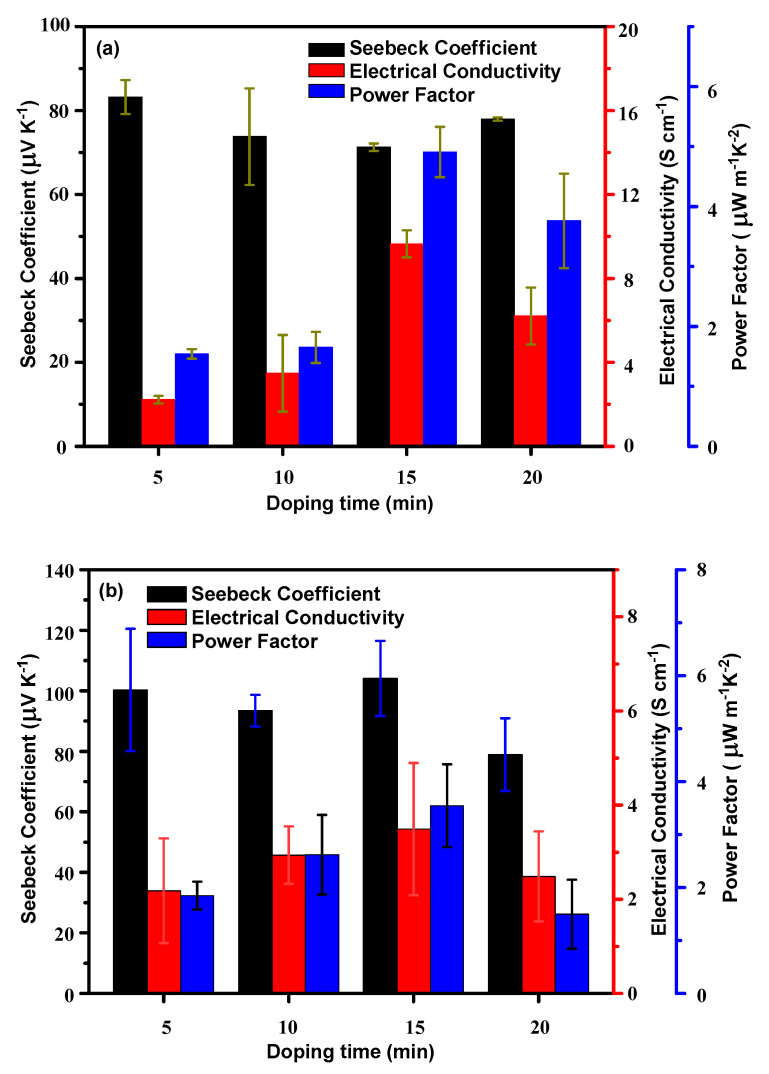
Thermoelectric performance for **P1** (**a**) and **P2** (**b**) films with different doping times (immersed in the 0.1 M FeCl_3_/acetonitrile) at room temperature.

The electrical conductivities were performed as shown in Figure 6. The electrical conductivities of **P1** initially enhanced with increasing doping time and reached to 10.49 S cm^−1^ for the 15 min doping, whereas slightly decreased for doping with 20 min. A similar trend for the electrical conductivities is also observed when doping **P2** films, and the maximal conductivity of **P2** was determined to be 3.46 S cm^−1^ at 15 min doping, which is much lower than that of **P1**. These results indicated that the incorporation of the large size aromatic benzene ring is detrimental to the conductivity of the IDT-based copolymers. The Seebeck coefficients of both **P1** and **P2** only exhibited slight change with varying doping time, and the Seebeck coefficient of 104.65 μV K^−1^ was obtained for **P2** after doping with FeCl_3_ for 15 min, which is higher than that of **P1** (72.43 μV K^−1^) under the same doping condition and time, thus the maximal power factor was obtained as 4.92 μW m^−1^ K^−2^ for doping **P1** films for 15 min at room temperature, which is higher than that of **P2** (3.48 μW m^−1^ K^−2^) for the same doping time (15 min).

## 4. Conclusions

We systematically investigated the effect of different size-chains on the thermoelectric performance of IDT-based conjugated polymers. We found that the incorporation of the large size aromatic benzene ring to the side chain does not change the electronic features of the semiconducting polymers, but reduced the interactions of the intermolecular packing of the polymer backbones due to the formed shishi-kebba structure with the aromatic benzene ring perpendicular to the backbone. Therefore, the introduced conjugated side chain on **P2** has a negative effect on the electrical conductivities by comparing the thermoelectric performances of **P1**. This work indicates that the alternation of the side chains of conjugated polymers has a great influence of the thermoelectric performance, and the investigation of the structure–properties relationships is a meaningful tactic to optimize the thermoelectric performance.

## Figures and Tables

**Table 1:** Molecular weights and thermal properties.

Polymer	*M_n_* (KDa)	*M_w_* (KDa)	PDI	*T_d_* (°C)	*T_g_* (°C)
**P1**	58.54	132.7	2.27	401	133
**P2**	87.11	166.3	1.91	392	203

## Data Availability

Not applicable

## Supplementary Materials

The following are available online: Figure S1: ^1^H-NMR spectrum of **P1** (up) and **P2** (down) (CDCl_3_, 600 MHz, room temperature), Figure S2: GPC curve of **P1**, Figure S3: GPC curve of **P2**, Figure S4: TGA curves of **P1** and **P2**, Figure S5: DSC curves of **P1** and **P2**, Figure S6: DFT calculations of **P1** and **P2** dimers, Figure S7: SEM and EDS images of doped **P1** and **P2**, Figure S8: AFM phase images of **P1** and **P2** before and after doping, Figure S9: Calculated intensities of *λ*_854_/*λ*_630_ for **P1** (black) and *λ*_856_/*λ*_620_ for **P2** (red).

## Sample Availability

Samples of the compounds **P1** and **P2** are available from the authors.
