# Supplementary material for: Effect of Side Chain Substituent Volume on Thermoelectric Properties of IDT-Based Conjugated Polymers
Source: Molecules. 2021 Feb 11;26(4):963. doi: 10.3390/molecules26040963 (PMC7918053; doi:10.3390/molecules26040963)
Supplement: Supplementary file 1 [file molecules-26-00963-s001.pdf]

Supporting Information

Effect of Side Chain Substituent Volume on Thermoelectric Properties of IDT-Based Conjugated Polymers

De-Xun Xie [1,2], Tong-Chao Liu [3], Jing Xiao [1,2], Jing-Kun Fang [4,*], Cheng-Jun Pan [3] and Guang Shao [1,2,*]

[1] School of Chemistry, Sun Yat-sen University, Guangzhou 510275, China; xiedx9@mail.sysu.edu.cn (D.-X.X.); xiaoj69@mail.sysu.edu.cn (J.X.)
[2] Shenzhen Research Institute, Sun Yat-sen University, Shenzhen, 518057, China
[3] Shenzhen Key Laboratory of Polymer Science and Technology, College of Materials Science and Engineering, Shenzhen University, Shenzhen 518060, China: liu21645@163.com (T.-C.L.); pancj@szu.edu.cn (C.-J.P.)
[4] School of Chemical Engineering, Nanjing University of Science and Technology, Nanjing, 210094, China
* Correspondence: shaog@mail.sysu.edu.cn (G.S.); fjk_003@163.com (J.-K.F.)

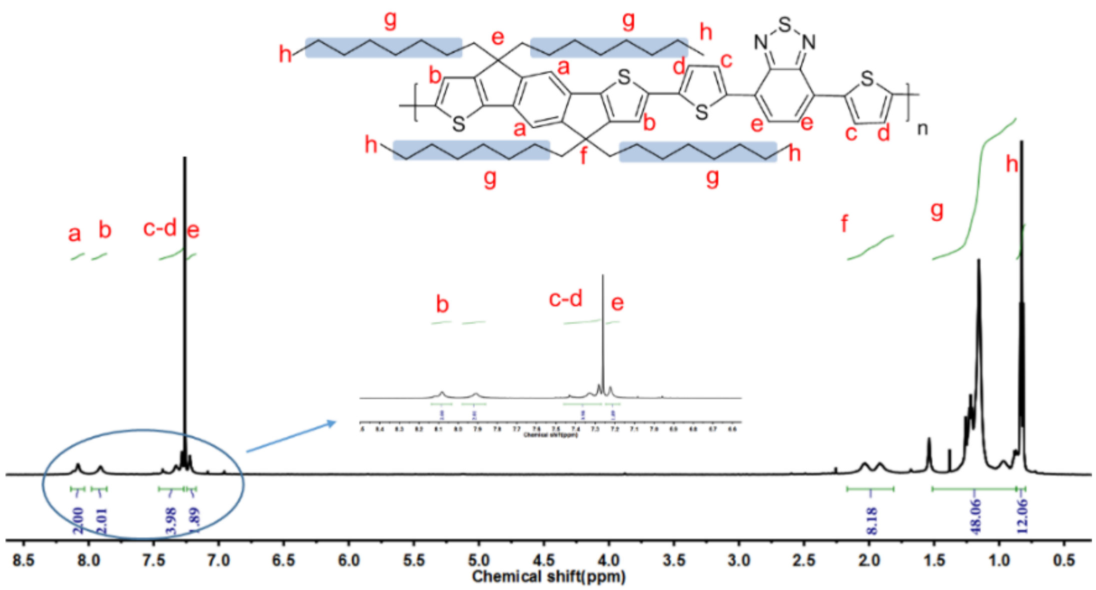

(**a**)

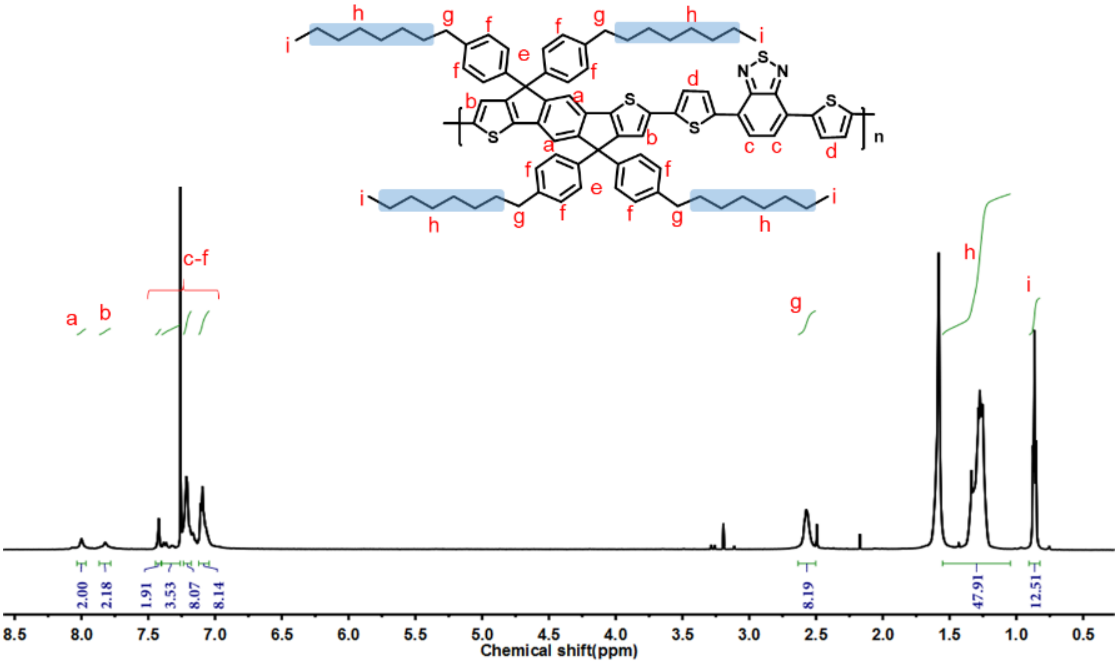

(**b**)

Figure S1. ^{1}H NMR spectrum of **P1**(**a**) and **P2** (**b**) ($CDCl_3$, 600 MHz, room temperature).

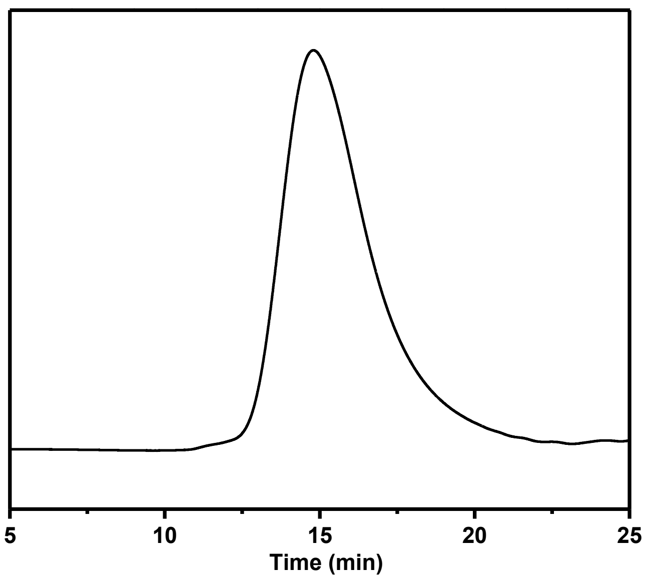

Figure S2. GPC curve of **P1**.

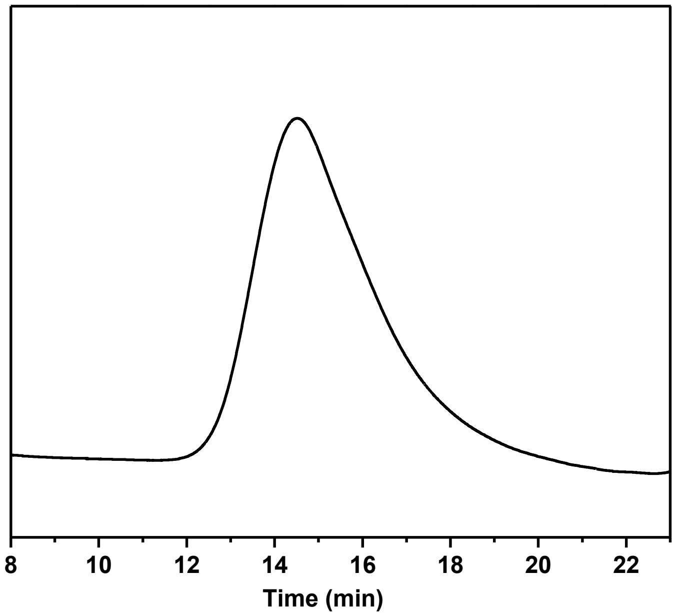

Figure S3. GPC curve of **P2**.

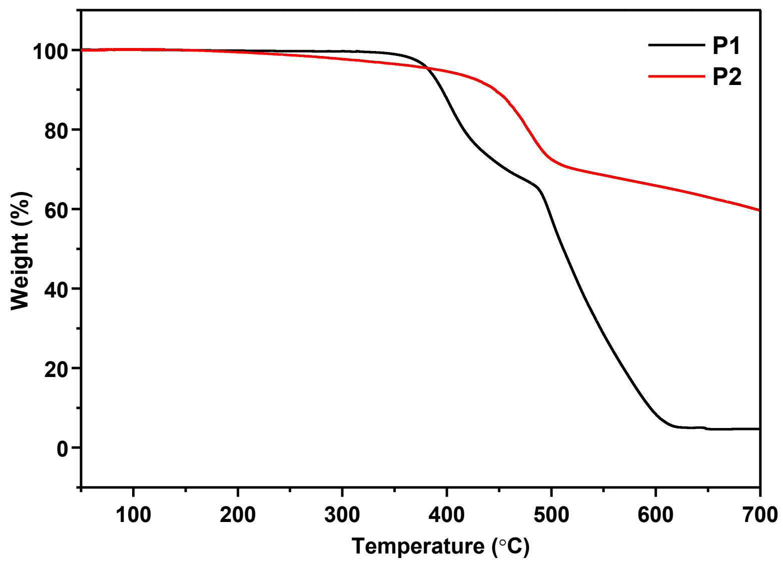

Figure S4. TGA curves of **P1** and **P2**.

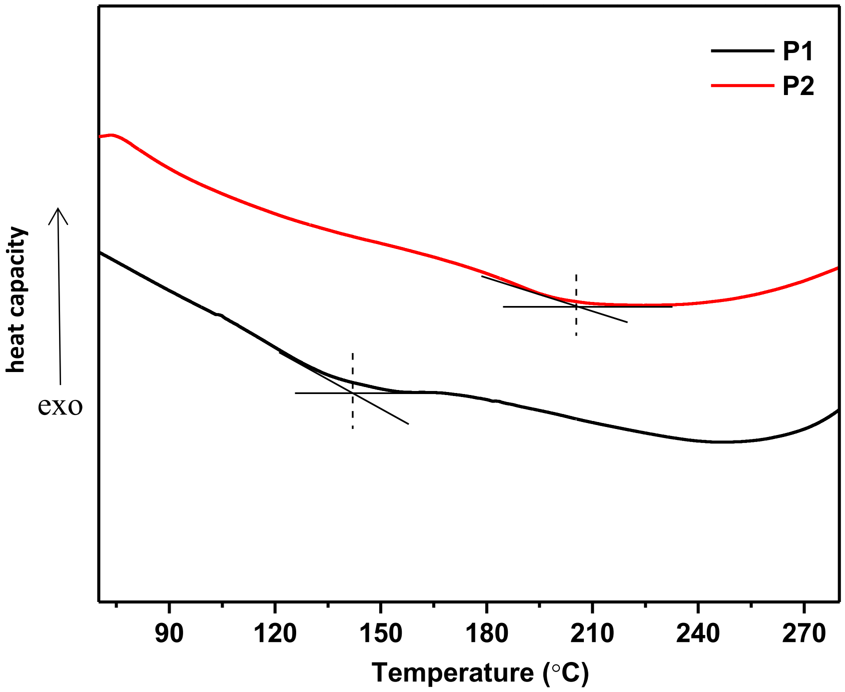

Figure S5. DSC curves of **P1** and **P2**.

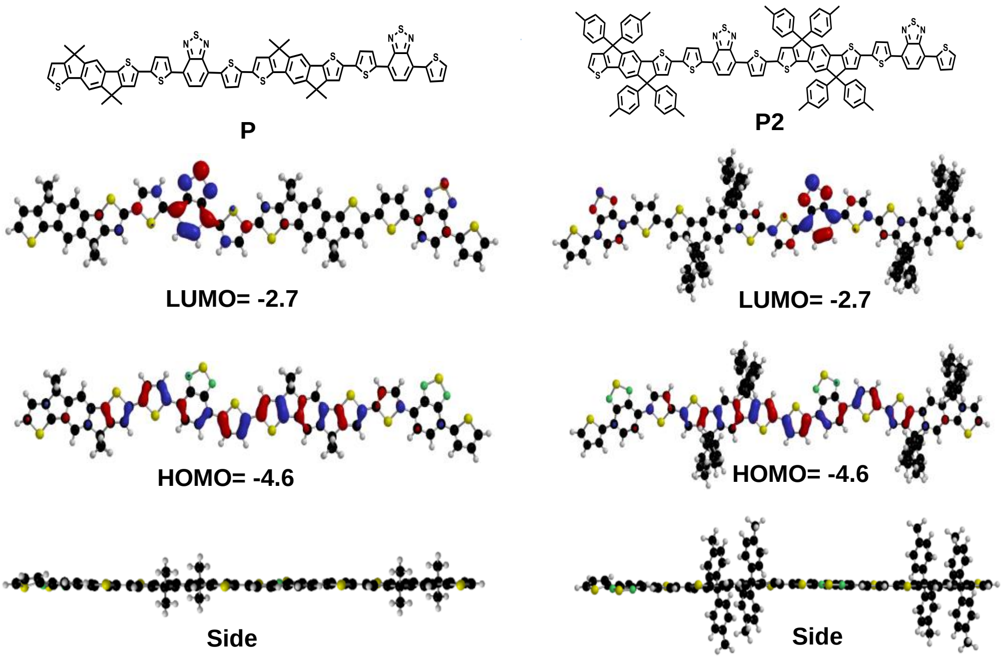

Figure S6. DFT calculations of **P1** and **P2** dimers.

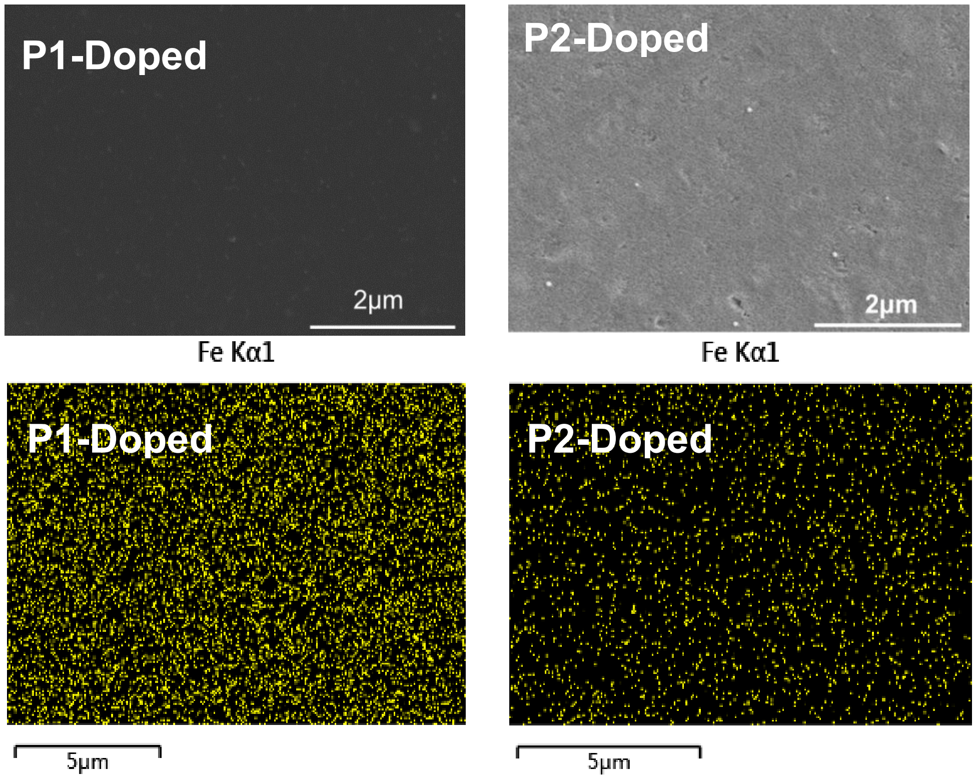

Figure S7. SEM and EDS images of doped **P1** and **P2**.

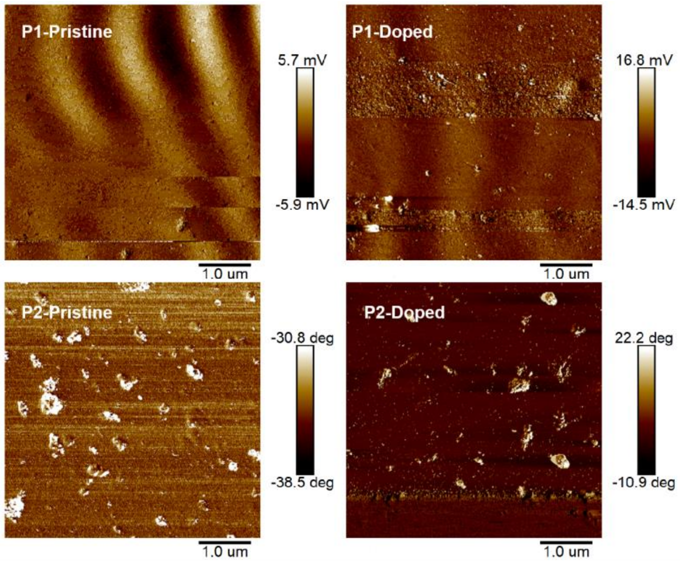

Figure S8. AFM phase images of **P1** and **P2** before and after doping.

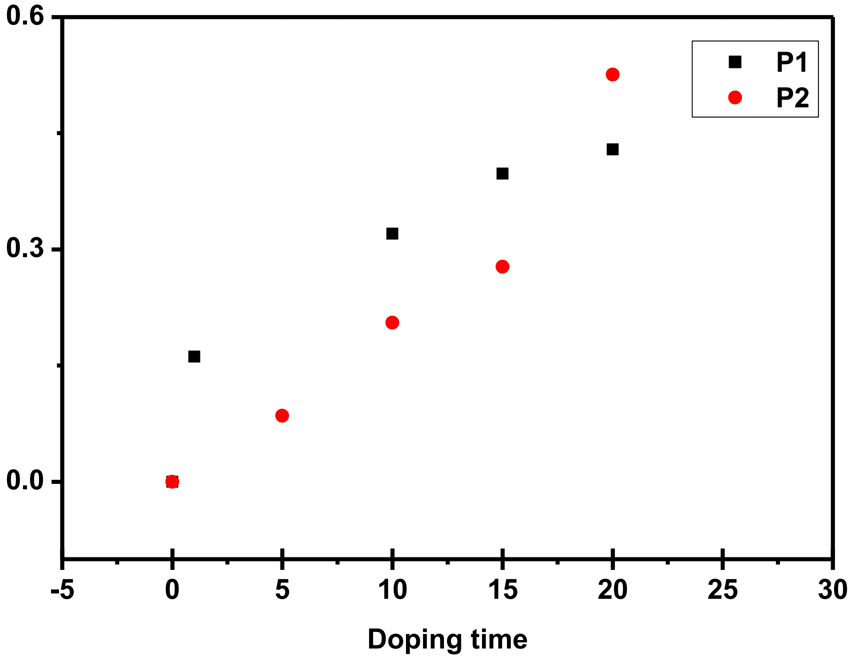

Figure 9. Calculated intensities of $\lambda_{854}/\lambda_{630}$ for **P1** (black) and $\lambda_{856}/\lambda_{620}$ for **P2** (red).
